# AN ANALYSIS OF C-REACTIVE PROTEIN AND COGNITIVE FUNCTION IN OLDER CHINESE ADULTS

**DOI:** 10.1093/geroni/igz038.3337

**Published:** 2019-11-08

**Authors:** Xi Pan, Ye Luo

**Affiliations:** 1 Texas State University, San Marcos, Texas, United States; 2 Clemson University, Clemson, South Carolina, United States

## Abstract

Recent evidence suggests that inflammatory markers may reflect cerebral disease mechanisms related to dementia. However, whether and how inflammation is associated with cognitive function in older Chinese is unknown. The current study is to analyze the association between high-sensitivity C-reactive protein (CRP), an indicator of chronic inflammation, and domain-specific cognitive function (i.e., mental status, memory, and overall cognition) among older adults aged 65 years and over in China. Data of the study (n=2,438) were selected from the 2015 China Health and Retirement Longitudinal Study (CHARLS) and were analyzed using linear regression models. Results of the study show that for every 1.00 mg/L increase in CRP, the scores of mental status and overall cognition both decreases by 0.03 unit and higher levels of CRP are observed in men than women. The findings might be useful in interpreting the biological mechanisms of dementia and add value in dementia prevention and health promotion for the general older population.

